# OntoDas – a tool for facilitating the construction of complex queries to the Gene Ontology

**DOI:** 10.1186/1471-2105-9-437

**Published:** 2008-10-16

**Authors:** Kieran O'Neill, Alexander Garcia, Anita Schwegmann, Rafael C Jimenez, Dan Jacobson, Henning Hermjakob

**Affiliations:** 1 Central Node, National Bioinformatics Network, Golf Park, Raapenberg Road, Cape Town, South Africa; 2 School of Computer Science, University of KwaZulu-Natal, King Edward Avenue, Pietermaritzburg, South Africa; 3 Institute of Infectious Diseases and Molecular Medicine, University of Cape Town, Cape Town, South Africa; 4 Faculty of Linguistics and Literary Sciences, University of Bremen, Bremen, Germany; 5 EMBL Outstation-European Bioinformatics Institute (EBI), Wellcome Trust Genome Campus, Hinxton, Cambridgeshire CB10 1SD, UK

## Abstract

**Background:**

Ontologies such as the Gene Ontology can enable the construction of complex queries over biological information in a conceptual way, however existing systems to do this are too technical. Within the biological domain there is an increasing need for software that facilitates the flexible retrieval of information. OntoDas aims to fulfil this need by allowing the definition of queries by selecting valid ontology terms.

**Results:**

OntoDas is a web-based tool that uses information visualisation techniques to provide an intuitive, interactive environment for constructing ontology-based queries against the Gene Ontology Database. Both a comprehensive use case and the interface itself were designed in a participatory manner by working with biologists to ensure that the interface matches the way biologists work. OntoDas was further tested with a separate group of biologists and refined based on their suggestions.

**Conclusion:**

OntoDas provides a visual and intuitive means for constructing complex queries against the Gene Ontology. It was designed with the participation of biologists and compares favourably with similar tools. It is available at

## Background

### Information visualisation and ontologies for information integration

Biological ontologies have recently become a cornerstone for integrating information within the biomedical domain. Biological information is not only highly nested, interconnected and distributed but also heterogeneous in both semantics and syntax [1]. The problem of syntactic heterogeneity has been partially addressed by the development of systems such as the Sequence Retrieval Service (SRS) [2], Entrez [3], Distributed Annotation System (DAS) [4] and others which overcome heterogeneity in the structure and representation of data. Semantics has proven to be more difficult [5], as it deals not only with the meaning and interpretation but also with the context of the data – information. While both syntax and semantics are important obstacles to overcome when constructing knowledge discovery tools for biologists, additional aspects need to be considered. In this vein, and in parallel with the development of bio-ontologies, research from the field of information visualisation, which aims to provide cognitive support to users working with abstract information [6], has begun to be applied to the biological domain [7-9]. Both of these efforts aim to assist biologists in discovering new biological knowledge by overcoming the semantic heterogeneity in bioinformatics, providing semantic [5] or "higher level" [10] data integration.

Numerous bio-ontologies have been developed, the Gene Ontology (GO) being one of the most visible and widely used [11]. GO is a controlled vocabulary consisting of three distinct ontologies intended to describe the roles of genes and gene products in any organism [12]. GO has been used to annotate gene products in numerous model organisms, providing a controlled vocabulary of terms for these annotations as well as a formalisation of the relationships amongst them. Since a common set of terms exists, queries can be formulated to find gene products with similar functions across the databases annotated with GO. Furthermore, the hierarchy can be transited over, so that queries to find gene products which participate in the process of coagulation will return those participating more specifically in blood coagulation as well as those participating in the regulation of blood coagulation; this enables relationships amongst gene products to be discovered where they were not obvious from their immediate annotations. Finally, multiple term queries can be formulated to find highly specific sets of gene products sharing multiple functions. An example of such a query might be:

"Retrieve gene products that *participate in ***blood coagulation **and *are located in *the **extracellular space **and *have function ***protease inhibitor activity**."

Such queries can be executed using scripts, such as the Perl API to the GO MySQL database. However, both the task of constructing queries in a scripting language and the task of making sense of the results place high cognitive load on the user. These may be made accessible to bench scientists if they could be handled through a GUI.

### Query previews

Form-based interfaces for query construction present two problems: Firstly, there is no guarantee that the user will guess the name of a real ontology term [13], nor, since ontology terms themselves can be ambiguous, that a term found will represent what they had in mind [14]. For instance, there is no term for expressing the concept of "inhibition of phagocyte maturation" in GO, although such a term could certainly be used to describe such a process which is known to occur during *Listeria monocytogenes *infection of monocytes. Secondly, combinations of terms entered often generate zero-hit queries, or large result sets which users must browse through [13]; in both cases the result set may not match a user's expectations, or be representative of the entire search space. Combining the GO terms *blood coagulation *and *ommochrome biosynthetic process *in a query will result in an empty set, whereas a query consisting of *protein binding *and *cytoplasm *will return most of the genes in the dataset. Query previews, a technique from information visualisation which combines browsing with querying, offer a solution to these problems [13]: In query previews, the user is presented with additional terms which can be added at each step when formulating the query. To guide users when selecting from these terms, a summarised preview of the result of each potential new query is provided, often in the form of the number of results. Furthermore, by only displaying those options which will return results, zero-hit queries can be eliminated: This helps to avoid user frustration and reduces cognitive load by presenting users with fewer options [13]. These benefits have been realised in software for constructing ontology-based queries outside of the molecular biological domain: Flamenco [15] and the Drug Ontology Project for Elsevier (DOPE) browser [16] make use of query previews for queries over ontology-annotated data sets. Within bioinformatics, however, such an interface has not, to our knowledge, so far been created.

### Existing ontology-based query systems

When applied to biological ontologies, information visualisation has so far focused mainly on providing interfaces for browsing and finding individual ontology terms. Examples of such tools include AmiGO [17], QuickGO [18] and the Ontology Lookup Service (OLS) [19]. A few broader information integration tools, such as BioMart [20] and its web interface, MartView [21] provide the functionality for constructing ontology-based queries. Another tool, GViewer [22], enables the construction of ontology-based queries over the information stored in the rat (*Rattus norvegicus*) genome database [23]. However, neither of these tools was designed specifically with the purpose of facilitating ontology-based queries: BioMart is intended to be a generic data warehousing system [20], while GViewer focuses on providing a sophisticated genome-wide view of the results of an ontology-based query, but has a simple form-based interface for entering the query itself [22]. Outside of the biological domain, visual tools specifically aimed at facilitating the construction of ontology-based queries have been created, including the DOPE browser [16,24] and Flamenco [15]. A comparison of these tools is presented in table 1.

**Table 1:** Comparison of ontology-based query interfaces. This table shows a comparison of OntoDas with existing systems designed to facilitate ontology-based queries, using various criteria

**Criterion**	**AmiGO**	**MartView**	**GViewer**	**DOPE**	**Flamenco**	**OntoDas**
1. Problem domains:	Gene Ontology (25 K terms) to multi-species gene DB (2 M entries)	Gene Ontology (25 K terms), individual species gene DBs (30 K entries each)	Gene Ontology (25 K terms), several others (5 K each); rat gene and QTL data (9 K entries)	EMTREE ontology (50 K terms), custom literature DB (10 M entries)	image metadata thesauri (4 K terms each); image databases (35 K entries each)	Gene Ontology (25 K terms) to multi-species gene DB (2 M entries)

2. Types of queries:	single term only; narrowing by evidence code, species.	multiple terms, only one term, evidence code per ontology. Only one species per query	AND, OR, NOT over several ontologies; no species/evidence code narrowing	AND queries of any number of terms; no narrowing criteria	AND queries across any number of terms; only one term per orthogonal ontology; supplementation with keyword search	AND queries across any number of terms

3. Initial term finding:	forms, tree navigation	QuickGO browse, search, but no tie-in with MartView interface	no support	Form with intelligent term suggestion; tree navigation	keyword search, dynamic tree navigation	not yet implemented

4. Combination finding:	no support	no support	no support	valid combinations with first term shown, but limited support for 3 or more	all valid combinations with current query displayed, also size of result set (query previewing)	extensive; all valid combinations displayed, as well as size of result set (query previewing)

5. Display of results:	paged table, links to detail on each query, links to external information.	simplistic but configurable to be richer; spreadsheet export	highly visual SVG; no table but proprietary spreadsheet export	interactive, visual cluster map; problems with scalability	page-able table, links to detail on each entry, ability to construct new queries from annotations of entry	table with links out; paging and CSV download not yet implemented

6. User involvement:	minimal, though possibly via mailing list	no evidence of any	no evidence of any	usability evaluation post- development	multiple cycles of testing, re-development, evaluated against a baseline	extensive participatory design throughout the life cycle

7. Technologies:	web-based: Perl, MySQL	web-based: Perl, BioMart	web-based: Java JSP and Oracle 9i PL/SQL	Desktop-based: Java/Swing, ClusterMap, Sesame RDF store	Web-based: Python WebWare, MySQL, Java/Lucene optional	Web-based: Ajax (MochiKit and others), Python TurboGears, MySQL, web services

### Presenting OntoDas

Software that visually facilitates the construction of complex, ontology-based queries within bioinformatics would be useful [25]; OntoDas aims to fulfil this need. This paper is organised as follows: The implementation section presents existing software for this purpose in more detail, introduces Dasty2 [26], then shows how OntoDas was designed to meet this need. The design process is described and illustrated by means of a biological scenario. Additional visual considerations as well as aspects of the technical design of the OntoDas query execution web service are outlined here. This is followed by the results and discussion section, which summarises the novel functionality of OntoDas by comparing it to existing software, describes a usability evaluation exercise carried out with biologists, discusses insights arising from the development of OntoDas, and presents possibilities for future work. The final section of the paper contains a summary of the conclusions drawn.

## Implementation

**Figure 1:**
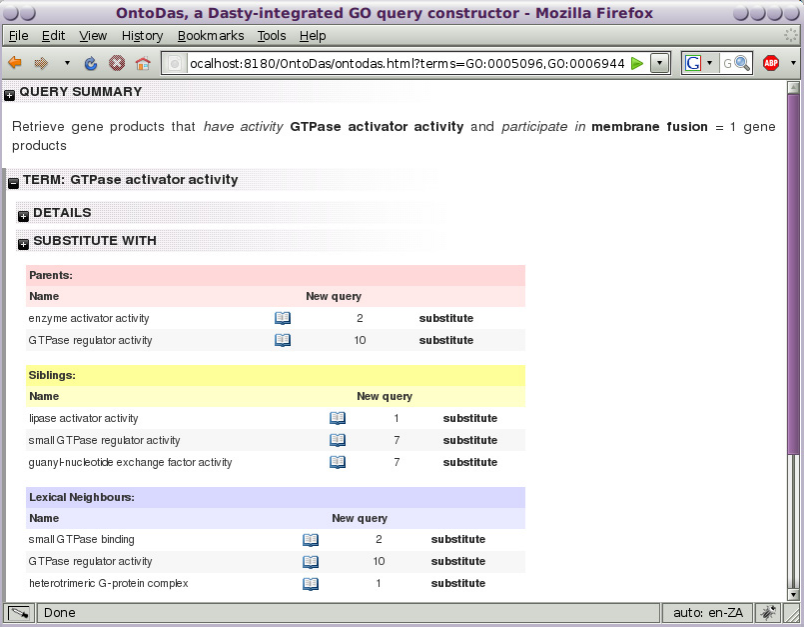
**Substitute term view**. The "substitute term" panel for the term *GTPase activator activity *in a complex query. Presented are the parents, siblings and lexical neighbours of the term, as well as the size of the queries which could be created by selecting any of these as a substitute for the term in focus. Substitution with *guanyl-nucleotide exchange factor activity *returns 7 gene products.

**Figure 2:**
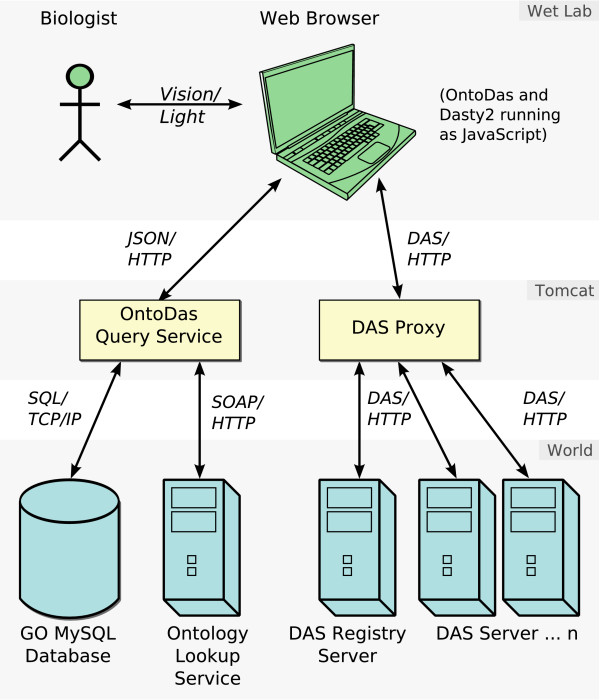
**OntoDas Architecture**. OntoDas has a three-tier architecture: The display tier consists of Ajax running within a web browser, being used by a researcher. The "business" tier runs on an Apache Tomcat web server, and provides query execution as well as DAS proxying. The final tier consists of external systems: The Ontology Lookup Service as well as various DAS services are accessed remotely via HTTP. The GO MySQL database is intended to be installed on a MySQL server on a local network with the Tomcat server.

OntoDas was created to be a web-based tool for constructing complex queries against the Gene Ontology. To ensure that it complies with the way in which molecular biologists work, design was carried out with the participation of a biologist who works with infectious diseases, particularly listeriosis. In addition, established information visualisation techniques were used to design an interface specifically designed to facilitate the construction and exploration of complex ontology-based queries. Technically, OntoDas was implemented as a JavaScript application, using web services to communicate with multiple servers and integrate the results in a single view. A web service was implemented around the MySQL version of the GO database to execute queries. The viewing of sequence annotations is provided via the inclusion of Dasty2. Finally, the Ontology Lookup Service is used to facilitate the browsing of query space via lexical similarity between terms (as shown in Figure 1).  This is summarised in figure 2.

### DAS and Dasty2

The design of OntoDas was partially influenced by that of Dasty2, the DAS client integrated into the system. The Distributed annotation system (DAS) provides representational state transfer (REST) style web service to several hundred databases running DAS servers worldwide. This enables transparent access to multiple repositories of biological annotations [4], allowing sophisticated visual information integration clients, such as Dasty2, to be built. Dasty2 not only provides a powerful, information-rich view of individual protein sequences, but also illustrates how a rich web client can be created to enable the integration of biological information from multiple sources using the Ajax (asynchronous Javascript and XML) paradigm [27]. As such, it provided both a useful extension to OntoDas for displaying information about gene products, as well as a model on which to base the OntoDas design.

### Participatory design of the interface

Given the uniqueness and complexity of the knowledge domain of molecular biology, a participatory design (PD) [28] approach was taken during the design of OntoDas. PD involves the users actively in the development of the tools or processes they use in their work; this is to ensure that the products designed meet the needs and work practices of the users [29]. This was done by conducting a series of interviews between the software engineer and the molecular immunologist to capture scenarios of use.

### Scenario: Investigating the inhibition of phagosome maturation by Listeria bacteria

**Figure 3:**
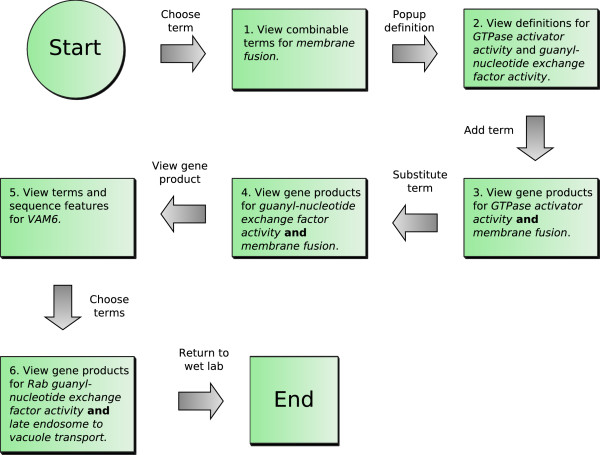
**Summary of the biological scenario workflow**. The biological scenario detailed in the paper is illustrated here. This workflow represents just one of many paths that a biologist could take when using OntoDas.

**Figure 4:**
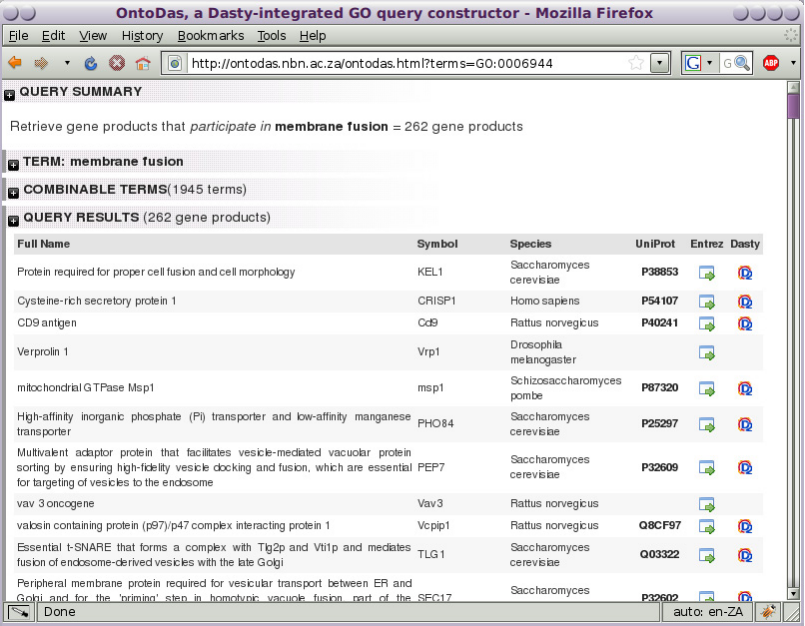
**Overview for a single-term query**. The OntoDas interface, showing a query involving just one term, *membrane fusion*. The query is quite general, returning 262 gene products. At this stage, the researcher is interested in narrowing the result set down using terms related to GTPase regulation.

**Figure 5:**
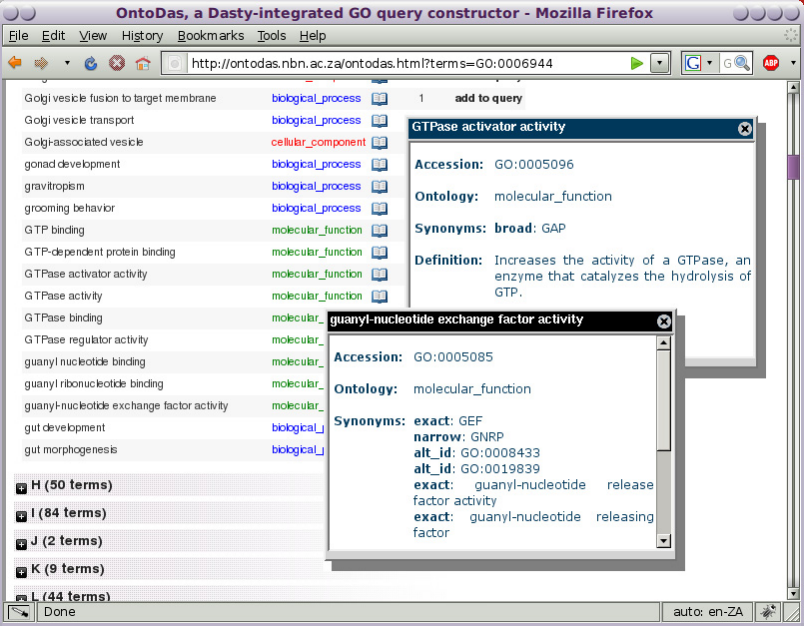
**Combinable terms panel**. The "combinable terms" panel of the *membrane fusion *query. The biologist has used the "group alphabetically" feature to look for terms beginning with "G". Clicking on the book icon next to the terms *GTPase activator activity *and *guanyl-nucleotide exchange factor activity*, they get details for both terms. The definitions pop up in a window, as well as synonyms, and provide the researcher with additional guidance as to which term best represents the concepts they are interested in.

**Figure 6:**
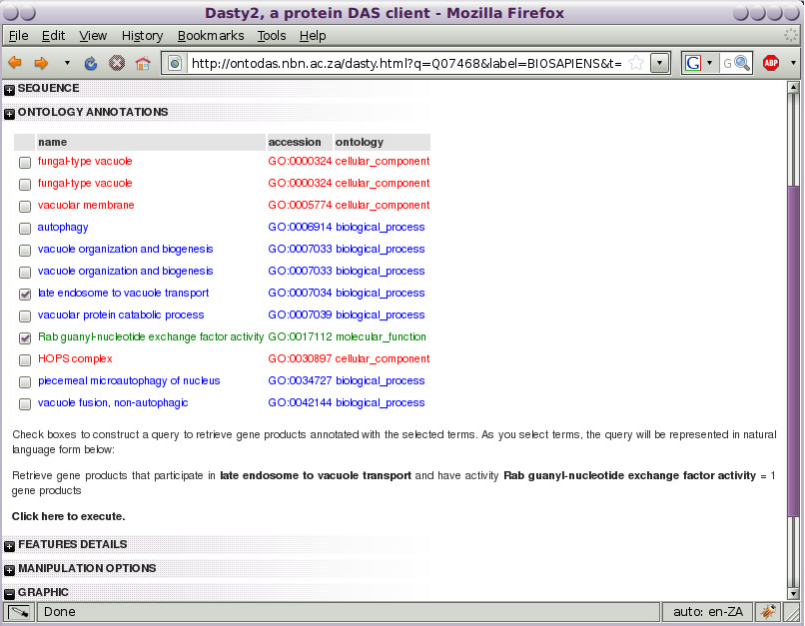
**Dasty2 additions**. Dasty2, showing the "Ontology Annotations" panel, which forms part of OntoDas. Shown here is the Dasty2 view for the protein encoded by the gene *Vam6*. Each ontology term used to annotate the given protein is displayed. Check boxes enable a combination of terms to be selected to construct a new ontology-term based query, the results of which are previewed below. Two terms have been selected for the final query.

**Figure 7:**
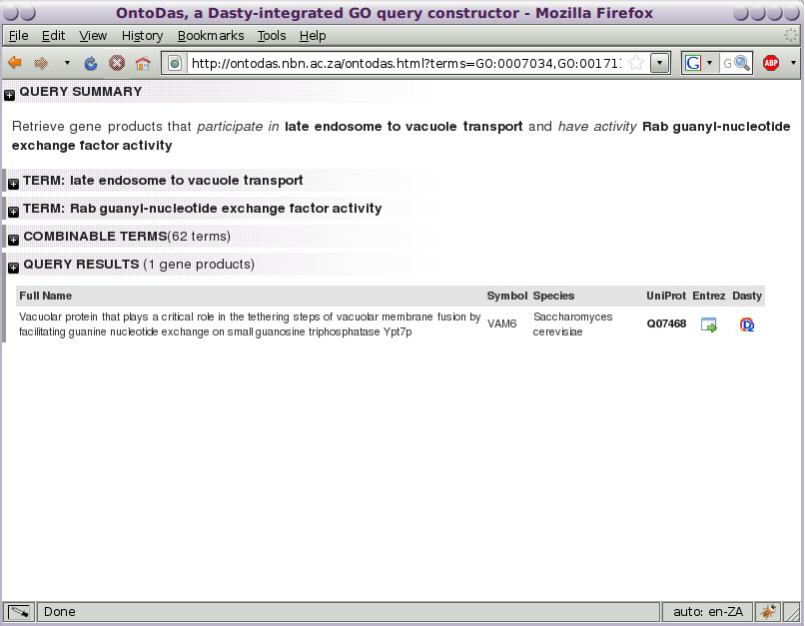
**Results of a focused query**. The overall OntoDas view, showing the narrowed down result set of just one gene product.

This section presents a scenario used in the design of OntoDas. The worflow of this scenario is illustrated in Figure 3, and the corresponding functionality illustrated in the form of screen shots in Figures 4, 5, 1, 6 and 7.

#### Background

The disease listeriosis is caused by the ubiquitous food-borne bacterium *Listeria monocytogenes *[30], and is of particular risk to immunocompromised individuals, such as those with HIV/AIDS or undergoing chemotherapy [31]. The bacterium invades host cells via phagocytosis (being enclosed in a membrane-bound vesicle called a phagosome) and the primary defence strategy of the host is to restrict the *Listeria *bacilli within phagosomes and promote fusion with lysosomes. The lysosome is an intracellular vesicle that is highly bactericidal and contains many hydrolytic enzymes which kill and digest the bacteria [32,33]. Phago-lysosome fusion can only occur once the pathogen-containing phagosome undergoes a sequential series of maturation events [33,34]. The process of phagosome maturation, phago-lysosome fusion and subsequent bacterial killing is regulated by the *Ras related protein 5a (Rab5a) *in response to *interferon-gamma *signalling [35,36]. When bound to GTP, *Rab5a *is biologically active and while carrying out it cellular functions, the GTP is hydrolysed into GDP. The inactive GDP-bound *Rab5a *becomes activated once more when the GDP molecule is replaced by a GTP molecule. The conversion between GTP-bound and GDP-bound states is facilitated by GTPase-activating proteins (GAPs) and guanine nucleotide exchange factors (GEFs). As part of its survival strategy, *Listeria *delays phago-lysosome fusion in order to "buy time" for itself so that it can escape from the phagosome via the activity of its secreted virulence factor, listeriolysin O [37]. *Listeria *delays phagosomal maturation by inhibiting the activity of *RAB guanine nucleotide exchange factor (GEF) 1 (Rabgef1)*, thereby preventing the exchange of inactive GDP-bound *Rab5a *for active GTP-bound *Rab5a *at the phagosomal membrane [38]. It is of interest to the researcher if there are other GEFs or GAPs involved in regulating phagosomal maturation and phago-lysosome fusion.

#### Scenario

1. The biologist begins with the term *membrane fusion*, since phagosomal maturation and phagolysosome fusion involve a series of membrane fusion events. The biologist is interested in terms pertaining to the regulation of *Rab5a*, which is a protein that has GTPase activity. The query for this single term is shown in Figure 4. By searching the combinable terms, the biologist sees that there are terms named *GTPase activator activity *and *guanyl-nucleotide exchange factor activity*.

2. To determine exactly how the Gene Ontology defines "GTPase activator activity" and "guanyl-nucleotide exchange factor activity", the researcher clicks on the book icon next to the term to pop up its definition. This is shown in Figure 5. Having determined that the GO term *GTPase activator activity *refers to the process of increasing the activity of a GTPase through the hydrolysis of GTP, and that *guanyl-nucleotide exchange factor activity *refers to stimulating the exchange of GDP by GTP nucleotides associated with a GTPase, they select *GTPase activator activity *to add to the query by clicking on the link marked "add to query".

3. This query returns only one gene product, *RABEP1*, which the researcher recognises as a GAP that interacts with *Rabgef1 *and stimulates *Rabgef1*-mediated guanyl-nucleotide exchange on *Rab5a *resulting in endocytic membrane fusion [39]. Indeed, in *Listeria*-infected macrophages of mice that are highly susceptible to *Listeria *infection, *Rabgef1 *expression was found by microarray to be down-regulated as compared to macrophages in resistant mice [40]. However, the researcher is interested in finding new gene products to develop new hypotheses, so, bringing up the substitution panel for *GTPase activator activity*, shown in Figure 1, they substitute *guanyl-nucleotide exchange factor activity*. In this view, they are also presented with lexical neighbours of the term which can be used in its place in the query.

4. On executing the query, the researcher finds seven results, including GEFs such as *RIMS1*, *VAM6*, *VAV3*, *SIZ*, *VPS39 *and *VPS41*. Surprisingly, the researcher notices that *Rabgef1 *is not annotated in the GO database to possess GEF activity even though its GEF activity has been proven experimentally [39] (and is implied in its name). For each of the gene products, the researcher follows the external links, and views their annotations in the Dasty view.

5. Dasty2 is used by the researcher to visualise the ontology terms used to annotate *VAM6*. Also available to them is the full Dasty2 view of sequence annotations for the protein from a large number of different databases. This is shown in Figure 6. In the list of ontology terms they see *Rab guanyl-nucleotide exchange factor activity *and *late endosome to vacuole transport*. This interests them, since *Listeria *interferes with *Rab5a *activity by modulating the activity of *Rabgef1*, a *Rab *GEF. They postulate that *Listeria *may also manipulate the activity of other *Rab *GEFs in order to hinder late endosome to vacuole transport and thereby prevent phagosome maturation and phago-lysosome fusion. Curious to find out what other proteins may be a target of *Listeria*, they check the terms *late endosome to vacuole transport *and *Rab guanyl-nucleotide exchange factor activity *to create a new query.

6. As shown in Figure 7, the query for these two terms is quite specific, returning only one gene product, *VAM6*. The biologist has identified one gene and generated the hypothesis that this gene may be negatively regulated by *Listeria *as part of the bacterium's survival strategy. The biologist can now test this hypothesis, generated through their interaction with OntoDas, in the wet laboratory.

### Additional visual considerations

OntoDas aims to provide as much useful information as possible, without overwhelming the user. To this end, OntoDas employs the following visual techniques: i) framing of queries in controlled natural language; ii) visual grouping and sorting of long lists of items; iii) the provision of grouping and sorting; iv) additional visual cues (colours, spatial arrangement, etc.) These are described in more detail below:

Queries are framed in controlled natural language so as to make them easier to understand. This representation uses phrases based upon the formal relations proposed by Smith *et al *[41]. The phrase *"participate in" *is used to refer to terms from the biological process ontology, to express the relation *has participant*. The phrase *"are located in" *refers to terms from the cellular component ontology, to express the relation *has location*. For terms from the molecular function ontology, since no relation has been agreed on, the phrase *"have function" *is used, expressing a simple, non-specific property relation.

Cues providing "information scent" have been included to guide users when choosing queries. Information scent is defined as *"the (imperfect) perception of the value, cost or access path of information sources obtained from proximal cues" *[42]. OntoDas provides these cues firstly in the form of query previews, and secondly through complete term definitions available in popup windows for each candidate term to be added to a query. Full term definitions may be useful because term names themselves are often ambiguous, whereas their natural language definitions are more likely to ensure terms' appropriate interpretation [14].

Dynamic grouping and sorting of lists of items can help to reduce cognitive load on users when searching for specific items by organising the lists into smaller, ordered lists which can be more easily visually processed [6]. In the OntoDas interface, two long lists of items are present: the list of combinable terms, and the list of resulting gene products. Controls are provided for the grouping and sorting of items in these lists. Additionally, similar terms which can be substituted for a term already in the query are visually grouped into parents, siblings, children and lexical neighbours.

### The query execution service

The query execution service provides the bridge between the browser-based Ajax component of OntoDas and the GO database. It is a RESTful web service; under the base URL of OntoDas there is a /queryservice/ directory, under which can be found a set of web service methods mapped to sub-URLs. These can be invoked via HTTP GET requests, with parameters being passed in the URL. The results are encoded as JSON, which can be easily decoded from within the browser-based component. The API for this service is included as an appendix to this paper.

The service was implemented as a prototype in Python, using the TurboGears framework. The final version was implemented as a Java web application to be run on the Apache Tomcat server. Queries are executed against a MySQL database loaded with the GO-lite MySQL data, processed additionally by Java code, and encoded into JSON. Lexical similarity queries are computed by invoking the OLS SOAP service from within the Java code. This architecture is illustrated in Figure 2.

### Computing combinable sets of terms

**Definition 1 ***Given an ontology defined as a digraph consisting of a set of terms T and a set of edges representing the transitive inheritance relation I on T × T, the successor set S*_*t *_⊆ *T for any given t ∈ T is defined:*

*s*_*t *_∈ *S*_*t *_⇔ *tIs*_*t*_

**Definition 2 ***Given a successor set S*_*t *_*for a given term t, and given a set of instances, C of T, related by an instance relation R on C × T, the instance set C*_*t *_⊆ *C of t is defined:*

*c *∈ *C*_*t *_⇔ ∃*s*_*t *_∈ *S*_*t*_; *cRs*_*t*_

The GO MySQL database is provided with the transitive closure of the graph of ontology terms pre-computed. This can be used to compute *S*_*t *_for a term. From this set, *C*_*t *_can be computed using the relation *R*, represented within the database by the "association" table. OntoDas uses SQL statements of the following form to determine *C*_*t*_:

SELECT DISTINCT association.gene_product_id FROM term INNER JOIN graph_path

ON (term.id = graph_path.term1_id) INNER JOIN association ON

(graph_path.term2_id = association.term_id) WHERE term.id = 12345

**Definition 3 ***Given a set of ontology terms *{*t*_1_, *t*_2_, ⋯}, *and their respective instance sets, C*_*t*1_, *C*_*t*2_, ⋯, *the set of common instances, C*_*c *_*for those terms is:*

*C*_*t*1 _∩ *C*_*t*2 _∩ ⋯

To compute *C*_*c*_, OntoDas computes *C*_*t *_for each term, then uses a standard set library to intersect these sets. This is the "result set" returned to the user via the OntoDas web user interface.

To implement query previewing, it is necessary to ensure that when a user is presented with terms which can be added to a query, they are only presented with those terms which will return a result. More formally:

**Definition 4 ***Given a common set of instances C*_*c*_, *for a set of terms Tq {t*_1_, *t*_2_, ⋯}, *the set of combinable terms, T*_*c *_*for T*_*q *_*can be defined:*

$$t_{c}\in T_{c}\Leftrightarrow C_{t_{c}}\cap C_{c}\neq \{\}$$

Or, equivalently:

$$t_{c}\in T_{c}\Leftrightarrow \exists c\in C_{c}|c\in C_{t_{c}}$$

**Figure 8:**
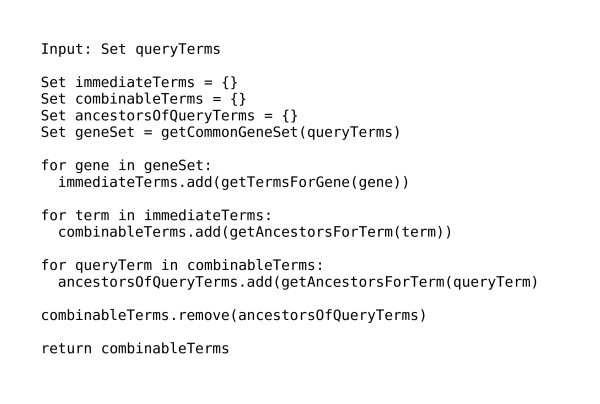
**Pseudocode showing how OntoDas computes combinable terms**. As described in the text, OntoDas works backwards from the result set of the current query to determine all of the terms which can be combined with it to produce a query with a non-empty result set. Ancestors of the query terms themselves are excluded, since adding them to the query would be redundant.

A simple way to compute the *T*_*c *_would be to combine *T*_*q *_with each other term in the ontology, then to test for each new query whether the result set was empty. However, this way could be extremely inefficient, as *C*_*c *_would need to be computed around 25 000 times. A more efficient way would be to work backwards from *C*_*c *_for *T*_*q*_, since every term used to annotate a gene product in *C*_*c *_must at least produce that gene product when combined with *T*_*q*_, and hence must be in *T*_*c*_. Furthermore, due to the transitivity of the inheritance relation, every ancestor of each of those immediate terms must also be in *T*_*c*_. Furthermore, since every member of *T*_*c *_must be either directly or transitively related to at least one of the gene products in *C*_*c*_, the complete set of all the terms immediately annotating those gene products, and their ancestors, is equivalent to *T*_*c*_. Pseudocode describing how OntoDas computes *T*_*c *_in this way is shown in Figure 8.

## Results and discussion

### OntoDas itself

OntoDas is a graphical web-based tool for constructing queries against the Gene Ontology database. OntoDas helps biologists to formulate and refine queries involving multiple ontology terms. By using query previews, all queries constructed within OntoDas are guaranteed to return at least one gene product. OntoDas improves on existing form-based interfaces by providing visual cognitive support to biologists when constructing queries. Finally, OntoDas was designed in collaboration with biologists, ensuring that the way in which biologists work was a prominent factor in the design.

### Evaluation of the finished tool

The tool was evaluated with a group of five domain experts (laboratory biologists). The biologists were confronted with a simple scenario: building a query and executing it – the query had to contain at least one GO term. The goal of the exercise was to understand how domain experts were building the query, how they were using the interface and how the interface could be improved.

From the exercise it was clear that OntoDas simplifies the process of building conceptual queries. The use of ontology concepts allowed the biologists to build queries that required more visualization tools as well as more cognitive support not only to retrieve the information but also to manipulate results. In this respect OntoDas was found to be better suited to this task than other existing tools. Nevertheless, the biologists requested further features, including:

• building of queries using relationships amongst terms (such as "is next to" for anatomy terms)

• reducing the amount of information displayed in the Dasty2 view (the biologists found they were scrolling through very long lists)

• tabbed views to allow results from different queries to be compared

An important outcome was the importance domain experts placed on features to facilitate viewing and organising information. Although OntoDas provides features to fulfil this, the use of even more sophisticated techniques could aid biologists in accessing and integrating information.

During the session the biologists constantly requested lists of combinable terms along with a facility for specifying relationships. For instance, providing the anatomical term "skull" then requesting a list of anatomical features both physically adjacent to the skull and combinable in a query with it. OntoDas cannot currently support this functionality because the ontology it works with (GO) relies on an *IS_A *hierarchy and a limited set of *PART_OF *relations. The same is true of most bio-ontologies, which similarly only represent taxonomic relations [10]. This supports the idea that more expressive relations, for instance those described by Smith *et al *[41], would be useful inclusions to bio-ontologies. A noteworthy observation during the evaluation was that the biologists constantly demanded substantially more expressivity from both the tool and the ontologies than is currently available.

In summary, the facility provided by OntoDas, to build conceptual queries aided biologists when carrying out the process of biological information integration. Nevertheless, once biologists began using OntoDas, they produced requirements for more sophisticated tools to carry out more complex queries, and provide greater facilities for visual data manipulation. This suggests that there is room in the future both for more expressive bio-ontologies and for more sophisticated visual tools which leverage these.

### Comparison with similar tools

As summarised in table 1, five tools have been selected as being sufficiently related to OntoDas for a meaningful comparison to be made. Within the molecular biological domain, AmiGO [17], MartView [21] and GViewer [22] were selected. All of these tools provide some querying capability for bio-ontologies, although only the latter two allow multiple term queries. All provide basic form-based interfaces to this functionality, without narrowing down the options presented as the query is built, as OntoDas does. Consequently, all are susceptible to the problems of finding both syntactically correct and semantically combinable terms, which OntoDas overcomes. Furthermore, no usability engineering was carried out on these interfaces, as was on OntoDas. Outside the domain, both DOPE [43] and Flamenco [43] do overcome these difficulties, but neither are applied to molecular biological ontologies. Also, although extensive usability work was carried out during the development of Flamenco, both the target users (visual artists) and the information being queried (images and their metadata) lie far outside of the domain of molecular biology. OntoDas represents the application of best practices established by DOPE and Flamenco within the molecular biological domain.

### Limitations

Although OntoDas supports most combinations of terms, two general exceptions exist: that of very general terms, combinable with nearly every term in GO and that of very specific terms which have not yet been used to annotate any gene products. It is unlikely that these will be of direct interest; nevertheless a future improvement to OntoDas may be to investigate ways in which these extreme cases could be used as starting points for queries.

Another limitation lies in the performance for queries in which around 1,000 or more combinable terms exist. Potential solutions include lazy loading of panels, and more server-oriented paging, as well as server-side optimisations such as caching and executing queries natively within MySQL via stored procedures. All of these measures are under consideration for future work.

### Future work

In addition to the improvements suggested above, the following are under consideration for future work: Additional web services such as the protein identifier cross-reference service (PICR) [44] may provide a greater range of links to other databases for individual gene products. The user interface can be further improved in collaboration with biologists. Extension of OntoDas to work with data annotated by multiple ontologies, such as those used in the mouse [45] and rat [46] genome databases is under consideration. Finally, extension of OntoDas to enable the construction of queries using the "OR" and "NOT" operators (in addition to "AND", which is currently supported) may be useful.

## Conclusion

OntoDas makes use of query previews and visualisation techniques to provide visual support in the construction of complex biological queries over the Gene Ontology. It was developed with the participation of biologists and using a detailed biological scenario. OntoDas compares favourably with other tools and fills a previously open niche within the bioinformatics world. Future work includes performance enhancements, interface improvements and investigations of the application of OntoDas to other ontologies and data sets.

## Availability and Requirements

**Project Name: **OntoDas

**Project Home Page: **; 

**Operating System(s): **Cross-platform

**Programming Languages: **JavaScript and Java

**Other Requirements: **Web browser (for client), Apache Tomcat, MySQL (for server)

**License: **Apache Software License, version 2.0

**Any restrictions to use by non-academics: **none

## Authors' contributions

KON coded the software and carried out the majority of the theoretical background research. AS provided extensive biological insight into the requirements and design of OntoDas, and provided the biological scenario used in this paper. RJ was the main developer of Dasty2, and provided extensive assistance in integrating it with OntoDas. AG supervised the work, carried out the evaluation with biologists and provided extensive intellectual guidance. HH and DJ provided higher-level supervision.
